# Acquisition of extended spectrum β-lactamases during travel abroad—A qualitative study among Swedish travellers examining their knowledge, risk assessment, and behaviour

**DOI:** 10.3402/qhw.v11.32378

**Published:** 2016-11-01

**Authors:** Susanne Wiklund, Ingegerd Fagerberg, Åke Örtqvist, Kristina Broliden, Ann Tammelin

**Affiliations:** 1 Department of Infection Control and Hospital Hygiene, Stockholm County Council, Stockholm, Sweden; 2 Unit of Infectious Diseases, Department of Medicine, Solna (MedS), Karolinska, Institutet, Stockholm, Sweden; 3 Department of Health Care Sciences, Ersta Sköndal University College, Stockholm, Sweden; 4 Department of Communicable Disease Control and Prevention, Stockholm County Council, Stockholm, Sweden; 5 Center for Molecular Medicine, Karolinska University Hospital, Stockholm, Sweden

**Keywords:** Grounded theory, ESBL, antibiotic resistance, antibiotic-resistant bacteria, travel, qualitative method

## Abstract

**Background:**

Travel to foreign countries involves the risk of becoming a carrier of antibiotic-resistant bacteria, especially when the destination is a country with a high prevalence of this type of bacteria.

**Aim and methods:**

The aim of this study was to learn about the knowledge of antibiotic resistance, and the behaviour and risk-taking among travellers, who had become carriers of extended spectrum beta-lactamases (ESBL)-producing bacteria during travel to a high-prevalence country. A modified version of grounded theory was used to analyse 15 open interviews.

**Results:**

The analysis resulted in a core category: *A need for knowledge to avoid risk-taking*. Before the journey, the participants did not perceive there to be any risk of becoming a carrier of antibiotic- resistant bacteria. The low level of knowledge of antibiotic-resistant bacteria and transmission routes influenced their behaviour and risk-taking during their journey, resulting in them exposing themselves to risk situations. After their trip, the majority did not believe that their personal risk behaviour could have caused them to become carriers of ESBL.

**Conclusion:**

The participants’ lack of knowledge of antibiotic-resistant bacteria resulted in unconscious risk-taking during their journey, which may have resulted in becoming carriers of ESBL-producing bacteria.

Travel to high-risk countries involves the risk of becoming a carrier of antibiotic-resistant bacteria. This is especially true when travelling to countries with a high prevalence of this type of bacteria, where there is often also extensive use of antibiotics and poor sanitation (Gaarslev & Stenderup, 1985; Kennedy & Collignon, 2010; Meyer, Gastmeier, Kola, & Schwab, 2012; Struelens et al., 2010). Southeast Asia, India and neighbouring countries, as well as the Middle East, are areas with a high prevalence of antibiotic-resistant bacteria (Oteo, Pérez-Vázqueza, & Camposa, 2010; Pitout, 2010; Ruppé et al., 2015; Hassing et al., 2015). Increasing globalization, with the migration of people, animals, and food across national borders, increases the risk of the spread of antibiotic-resistant bacteria. The growth of international air travel makes it possible to travel around the world in only a matter of hours instead of weeks, which enables the rapid spread of infectious diseases across borders (Rogers, Aminzadeh, Hayashi, & Paterson, 2011). Of more than 1 billion annual travellers, about 300 million visit high-risk areas and more than 20% return as new carriers of resistant intestinal bacteria (Kantele et al., 2015). A new type of traveller that is increasing is the “medical tourist.” Patients travel internationally for various procedures, such as cosmetic surgery, fertility treatment, or an organ transplant. The treatment centres are often located in countries with a high rate of antibiotic-resistant bacteria (Rogers, Aminzadeh, Hayashi, & Paterson, 2011).

Intestinal bacteria like *Escherichia coli, Klebsiella pneumoniae* and related species (Enterobacteriaceae) are some of the most common causes of infectious disease, including urinary tract infection, postoperative infection, and septicaemia, among others. Extended spectrum beta-lactamases (ESBL) are a group of enzymes that can be produced by species belonging to Enterobacteriaceae. The enzymes have the ability to break down antibiotics belonging to the beta-lactam group (penicillins, cephalosporins) leading to infections caused by ESBL-producing Enterobacteriaceae, which often have to be treated with carbapenems, a group of beta-lactam antibiotics that has a wide antibacterial spectrum but cannot be administrated orally (Giske & Tängdén, 2008; Woerther, Burdet, Chacaty, & Andremont, 2013).

The community carriage of ESBL is increasing rapidly in the world. The regions with the highest carriage rates are the Western Pacific, with more than 60% reported from Thailand, Eastern Mediterranean, and Southeast Asia (Woerther, Burdet, Chacaty, & Andremont, 2013). Also in Sweden the rate of persons carrying ESBL-producing bacteria has increased (Folkhälsomyndigheten, 2014). Travel to high-prevalence countries is probably contributing to this increase, and three Swedish studies have shown that 24–31% of persons travelling to such countries acquired ESBL-producing bacteria in faecal flora during their journey (Östholm-Balkhed et al., 2013; Tängdén, Cars, Melhus, & Löwdin, 2010; Tham et al., 2010). Among Swedish healthcare students travelling for pre-clinical and clinical courses abroad, especially to the South East Asia region, 35% of them became carriers of ESBL-producing bacteria during their stay abroad (Angelin et al., 2015). It is likely that the ESBL-producing bacteria most often are acquired from food, for example, vegetables and fruit washed in water contaminated with faecal bacteria (Gaarslev & Stenderup, 1985; Kennedy & Collignon, 2010; Meyer, Gastmeier, Kola, & Schwab, 2012; Struelens et al., 2010). The role of polluted water as a major reservoir for community-acquired ESBL has also been well documented. Poor access to drinking water, poverty, and a high population density are driving forces for faecally–orally transmitted microorganisms such as ESBL (Kennedy & Collignon, 2010). To prevent and reduce ESBL carriage in community populations, hand-washing has proved to be an effective method but, despite the low cost of soap, washing still remains inadequate in many parts of the world (Folkhälsomyndigheten, 2014). Another additional factor for an acquisition of ESBL during travel is the use of antibiotics. In a study by Kantele et al. (2015), 67% of 430 Finnish travellers got travellers’ diarrhoea during their trip to high-risk areas for ESBL. The overall antibiotics use among the travellers was 15% (66/430), and in 79% (52/66) of the cases they were used for travellers’ diarrhoea. Travellers’ diarrhoea and the use of antimicrobials for treating this condition were proved to be independent risk factors for acquiring ESBL during the trip. In a previous study, we found that a group of Swedish travellers to high-prevalence countries had large gaps in their knowledge and understanding of antibiotic-resistant bacteria and the risk of becoming a carrier before they started their journey. The travellers were ignorant of and unconcerned about antibiotic resistance, had a tendency for distancing themselves from the problem, and did not perceive that there was any major risk of becoming a carrier of resistant bacteria during their journey (Wiklund et al., 2015). However, there is no previous study of the knowledge of antibiotic resistance, behaviour, and risk-taking among travellers who actually have become carriers of ESBL-producing bacteria during such a journey.

## Methodology

The aim of this research was to learn about the knowledge of antibiotic resistance, and the behaviour and risk-taking among travellers, who have acquired a carriage of ESBL-producing bacteria during a trip to a high-prevalence country. The main concern was whether it was likely that the level of knowledge of antibiotic resistance had influenced some of the travellers’ behaviour and risk-taking during their journey. As this is a research area where there is no previous knowledge, grounded theory was selected as an appropriate scientific qualitative method. Grounded theory is a specific methodology developed by Glaser and Strauss (Glaser, 1978; Glaser & Strauss, 1967) for the purpose of building theory from data (Charmaz, 2006; Corbin & Strauss, 2008). The method has its roots in symbolic interactionism and includes the idea that meaning is constructed and changed within interactions between people. People act towards things based on the meaning those things have for them and these meanings are derived from social interaction and modified through personal interpretation (Birks & Mills, 2015; Denzin & Lincoln, 2005; Jeon, 2004). The basic principles of grounded theory are theoretical sampling, constant comparisons, theoretical sensitivity, and saturation. Theoretical sampling is used to reach saturation and is guided from the emerging categories. Constant comparison includes a constant comparison of raw data and emerging categories during the entire analysis process. Saturation is reached when new interviews do not bring additional information into the emerging categories (Charmaz, 2006; Corbin & Strauss, 2008; Glaser, 1978; Glaser & Strauss, 1967).

### Sample selection and recruitment

This was a retrospective study among Swedish travellers. The participants were recruited in collaboration with a clinic for travel medicine and vaccinations in Stockholm where many travellers go for vaccinations and advice prior to travel. Based on the principles of grounded theory of maximum variation in sampling (Glaser, 1978; Glaser & Strauss 1967), our initial purpose for the sampling recruitment was to get travellers from diverse backgrounds, age, and gender. During the period May–December 2013, people who planned to travel to India and neighbouring countries, Southeast Asia, North Africa, or the Middle East were asked to participate in a study led by Vading et al. (2016). Samples for ESBL were taken before and after travel. In a questionnaire the travellers were asked if they were willing to participate in an interview after their trip if the sample was found to be positive for ESBL when they returned. The first 26 travellers who accepted the request and who had become carriers of ESBL during their journey were contacted by the research team asking if they still agreed to participate. Written information with informed consent was delivered to their homes by ordinary mail. A reminder was sent out to those who did not respond. Fifteen of the twenty-six ESBL-positive persons agreed and were included in the study. The study group consisted of 12 women (mean age 52 years, range 35–69) and 3 men (mean age 52 years, range 29–67). Of these, 4 had travelled to Southeast Asia, 10 to India or neighbouring countries, and 1 to North Africa (Table 1). In total 11 persons did not choose to participate in the study for unknown reasons.

**Table 1:** Age, gender, and destination among participants.

	Women (*n*)	Men (*n*)	Total (*n*)	Mean age women (range)	Mean age men (range)	Mean age total (range)
Southeast Asia	3	1	4	52 (35–65)	67	55.8 (35–67)
India with neighbouring countries	8	2	10	58.1 (38–69)	44 (29–59)	55.3 (29–69)
North Africa	1	0	1	51	51	51
Total	12	3	15	51.8 (35–69)	51.7 (29–67)	55.1 (29–69)

### Data collection and data analysis

An open-ended, audio-taped interview, lasting up to 60 min, was conducted with each of the 15 participants during the period January–April 2014. The interviews were conducted by the main author and the location of the interview was chosen by the participants. An interview guide with two open-ended questions was used; “You have during your trip abroad become a carrier of intestinal bacteria that are resistant to antibiotics and I wonder if you could tell me: about your trip—what have you experienced and what are your thoughts about that you now have a carriage of resistant intestinal bacteria during the trip?” and “Do you think that you have exposed yourself to any risks for acquiring a carriership of ESBL during the trip?” In addition to these questions, the participants were free to tell about all their experiences during their journey and had the opportunity to raise questions of relevance to them. Data collection and analysis were conducted simultaneously and continued until new interviews did not provide additional information. The interviews were transcribed verbatim, and memos such as emotional expressions and longer breaks were added to the text.

**Figure 1:**
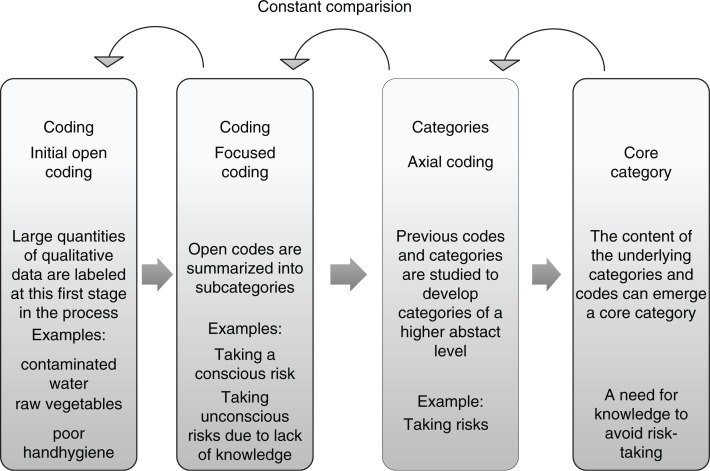
The process in the analysis of grounded theory (constructed by the main author, 2016).

The analysis started as soon as the first interview was completed and transcripted. A modified version of grounded theory according to Corbin & Staruss, (2008) was used for the analysis with the aim of studying social processes and interactions between people. Grounded theory combines data collection and data analysis in a constant comparative methodology. The material was read several times in order to identify similarities and differences. The first step in the analysis is the “open coding,” which means line-by-line reading, asking questions of “What is expressed here?” and “What is the meaning of this?” The content of the material was captured and reduced into substantive codes. Open codes with similar concepts or meaning were summarized into five emerging subcategories. In an axial coding process, these subcategories were related to each other, and two categories of a higher abstract level were created. The constant comparison method was used during the whole process to ensure that emerging codes and categories remained grounded in data. Interviews were re-analysed in light of new data emerging during the process. A coding paradigm according to Corbin and Strauss (2008) was used describing the stages causes/conditions—actions/interaction—consequences. Finally, a “core category” was identified, describing what the study was all about and reflecting the contents of the underlying categories (Figure 1).

### Ethical considerations

The study was conducted in accordance with the Helsinki Declaration. Ethical approval was obtained from the Regional Ethical Review Board in Stockholm (Reg. no. 2013/2:11). The integrity of participants was taken into account by ensuring both confidentiality and voluntary participation.

## Results

**Figure 2:**
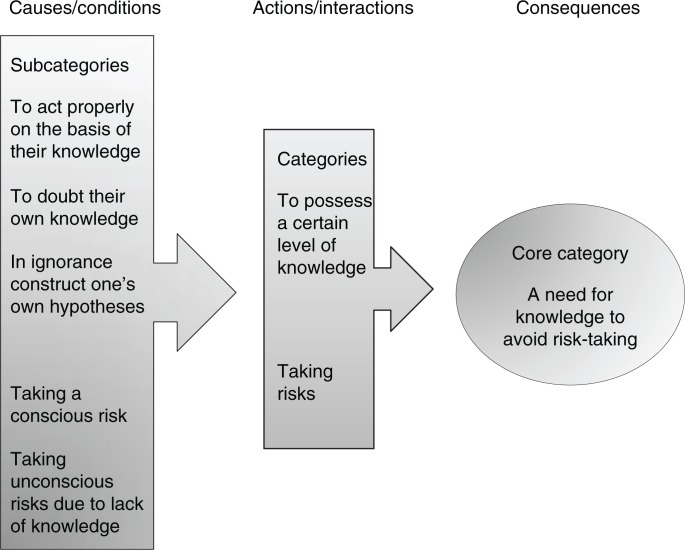
Subcategories, categories, and core category (constructed by the main author, 2016).

In the analysis process, a core category emerged: *A need for knowledge to avoid risk-taking*. The additional categories were further analysed, and the results are presented by category (Figure 2). During the trip abroad, the participants travelled around using cars, buses, trains, airplanes, and boats. They stayed in both urban and rural areas. The analysis indicates that the level of knowledge about antibiotic resistance affected their behaviour and risk-taking during their journey. The results showed that there is a need for more information to improve knowledge among travellers about how to reduce the risk of becoming a carrier of antibiotic-resistant bacteria during travel abroad.

### To possess a certain level of knowledge

#### To act properly on the basis of their knowledge

The travellers were either ignorant of or had a low level of knowledge of antibiotic resistance, antibiotic-resistant bacteria, and transmission routes. A few travellers showed some knowledge about the importance of clean water, hot food, and hand hygiene, but they were in minority.

Yes, I did think about washing my hands, before and after eating … and when I visited

the toilet I washed my hands carefully with soap and water, and so, but … I don't know … even at home too, it's something I try to be careful about

These travellers used their knowledge about preventive measures and acted properly in accordance with their knowledge.

#### To doubt their own knowledge

The participants lacked in knowledge about antibiotic-resistant bacteria and the issue caused uncertainty among the travellers. Before their journey, the participants did not perceive there to be any risk of becoming a carrier of antibiotic-resistant bacteria. Some of them had never reflected on this possibility before. After the journey, there were doubts about the cause of the carriership of ESBL, transmission routes, and also concerns about the consequences for the future.

I don't know the cause of the carriership of ESBL, if it's caused by food or anything else, I don't know … probably by food, but I cannot point to any special occasion where I got it … and I have no symptoms from my stomach

The participants were convinced that a carriership of ESBL resulted in gastrointestinal symptoms as diarrhoea and did not understand the fact that they had become carriers without any symptoms.

#### In ignorance construct one's own hypotheses

Lack of knowledge led them to construct their own hypotheses about antibiotic resistance, transmission routes, and risk assessment. The travellers thought that the risk of becoming a carrier of antibiotic-resistant bacteria was lower in a civilized country, if they were staying at middle- or high-class hotels, and in cities or tourist areas. There were also thoughts that carriership was predetermined, that there was nothing that could be done to prevent it, and that antibiotic resistance was transmitted through the air.

I heard before the journey that many people who travel there get this type of resistant bacteria. I heard a figure of 80% who came back and have got it. So then you get it, and that's that

The travellers thought that cholera vaccine, pro-biotic bacteria tablets, and papaya could have a preventive effect. They also believed that if you became a carrier of antibiotic-resistant intestinal bacteria, you would have symptoms such as diarrhoea and vomiting. There were many opinions concerning the role of food, such as what to eat and how to eat in a safe way. Some thought it could be preventive to eat vegetables only, some that you should choose fish or chicken, whereas others believed that you should avoid fish and chicken. If the food looked fresh, they thought the risk to be lower, like when visiting a high-class or international restaurant. If the restaurant had a high turnover of people, the risk was also considered to be less.

And most days we visited very fine restaurants in India, so this could be seen as a lower risk. I never ate any food from snack bar or food trucks or simple restaurants

After their trip, the majority did not see that they had engaged in any personal risk behaviour that could have caused their new carriership of ESBL. The reason for their new carriership was unclear to them.

### Taking risks

#### Taking a conscious risk

A few of the travellers had some knowledge of antibiotic-resistant bacteria and transmission routes, but they ignored these risks and consciously exposed themselves to risk situations. They knew that clean water was important and they used bottled water to drink. On the other hand, they brushed their teeth in tap water and washed the dishes in dirty water despite being aware of the risk. They were aware of the risk of eating some food, for example, raw vegetables and fruit that could have been rinsed in contaminated water, but because they wanted to eat it they did so, including also food from buffets.

I actually wanted to eat some salad and I actually wanted to try a little of the sauce, so I did. I was aware that this was on the “no-no” list, but I actually wanted to eat these things.

They knew that they should avoid the use of unnecessary antibiotics, but when they became ill with gastrointestinal symptoms, such as diarrhoea or vomiting, they took antibiotics without consulting a physician.

“And then the pharmacist picked up some of his drugs, and I asked what they were. I was so sick, so I thought, I'll take them anyway.”

They described situations where they became more and more careless as the trip went on. Some were invited to dinner with the locals, and the travellers perceived that this could be a risk as they did not know anything about the standard of hand hygiene of the hosts or how the meal was prepared. The meals included buffets and raw vegetables and fruit, but they felt it would have been difficult to question the hygiene standards of their hosts as they were invited guests.

#### Taking unconscious risks due to lack of knowledge

Overall, among the travellers there was a low level of knowledge of antibiotic-resistant bacteria and transmission routes, which influenced their behaviour and risk-taking during their journey. In their ignorance they exposed themselves to risks they were not aware of. Most had travelled to high-prevalence countries before and perceived the risk of carriership to be low if they behaved in the same way as on earlier trips. If the locals could carry on with their lives in that country, eating and drinking what they wanted, then travellers could do the same.

I have travelled a lot, lived in the countryside, eating and drinking as the people who live there, and I've never been sick. I've done it for many years and it has never bothered me. I have bought food in the market and been to the homes of poor rural families who invited me to meals prepared in terrible conditions

When it came to food they exposed themselves to risk. They ate raw vegetables and fruit and did not reflect on how and with what they had been rinsed. In the hotels they often ate from buffets, and they did not bother about the hygiene standard of the food. It was enough if the food looked fresh.

As far as food was concerned there was a lot of fish and it was freshly caught. It came directly from the sea, and therefore had not been stored in the wrong way … those things are important to me. Otherwise, I ate at restaurants, I always ate very fresh food. I ate quite a lot of fish and other sea food when it was available. Vegetables … yes, I had a lot … I ate shredded cabbage and stuff, cabbage and carrots, which they shredded—I saw that it was freshly grated

However, some travellers conducted no risk assessment for their choice of place for meals. They had their meals in various places, such as local or beach restaurants, canteens, or restaurants of higher standard. Some were invited by the locals for dinner in their private homes and they did not perceive that as a risk, because their hosts apparently could eat and drink what they wanted without becoming ill.

Because I lived and worked abroad for so long it cannot possibly be more dangerous in Thailand than in other countries I've been to. So therefore for three and a half weeks we had our meals at local restaurants. However, I was careful about seafood always being properly prepared. I know that it can be very dangerous with oysters, so I didn't have any at all, but I ate a lot of fish and sea food

In general, they drank bottled water, but they brushed their teeth in tap water and washed the dishes in contaminated water or rain water, unaware of the risk. They visited dirty public toilets where it was difficult to maintain good hand hygiene. If they developed diarrhoea or vomiting, they used antibiotics prescribed by a pharmacist or a physician, without any clear diagnosis, and without knowing what type of medicine they were taking.

A doctor came to our hotel because a man in the group developed a rather nasty infection. So we decided that we should also see a doctor, and then I thought he might examine me and check me out—I was feeling really bad. And he prescribed a load of tablets for me at once. He did not examine me as such, he just pressed my stomach and said, “Infection, infection,” and then he prescribed a lot of tablets … There were four different types … and then there were of course antibiotics as well, which I did not finish

They did not understand that antibiotic treatment could increase the risk of developing antibiotic-resistant bacteria. Some did not complete the course of antibiotic treatment, but stopped after a couple of tablets. It appeared that in some cases they had brought antibiotics with them from Sweden.

## Discussion

The analysis, performed according to grounded theory, generated a core category labelled *A need for knowledge to avoid risk-taking* with two additional categories and five subcategories, reflecting different aspects of the main concern. The aim of this study was to learn about the knowledge of antibiotic resistance, and the behaviour and risk-taking among travellers, who had become carriers of ESBL-producing bacteria during travel to a high-prevalence country. To the best of our knowledge, this is the first study examining the knowledge of antibiotic resistance, behaviour, and risk-taking among travellers abroad who have become carriers of ESBL-producing bacteria. We found that it was likely that the level of knowledge of antibiotic resistance had influenced some of the travellers’ behaviour and risk-taking during their journey. In general the travellers had a low level of knowledge or were ignorant about antibiotic resistance, antibiotic-resistant bacteria, and transmission routes, and in their ignorance they exposed themselves to risk situations of which they were unaware. Our findings are consistent with previous studies about risk perception among travellers, showing that many are not fully aware of the health hazards and do not always take appropriate safety precautions (Zimmerman, Hatterndorf, Blum, Nüesh, & Hatz, 2013).

One explanation why the travellers exposed themselves to risk situations could be that they did not feel that there was any risk, being unaware that the situation was unsafe. There is no commonly accepted definition for the term “risk”, and all risk concepts have one element in common, the distinction between reality and possibility. The term “risk” could be defined as the possibility that human actions or events lead to consequences that have an impact on what humans value (Renn, 1998). In this study, a risk assessment based on a lack of or limited knowledge may have resulted in a carriership of ESBL-producing bacteria.

Aro, Vartti, Schreck, Turtiainen, and Uutela et al. (2009) reported that younger travellers and those on holidays were willing to take more travel-related health risks than those who were older or on business trips. Holiday travellers more often posed risks related to “letting it all go” and looking for experiences that increased the risk of infectious diseases, food-related infections, and accidents. Our results were not similar to the study by Aro et al. The average age of our study group was 55 years and the group included both holiday and business travellers. Wynberg et al. (2013) stated that knowledge could affect risk assessment and behaviour and that professional pre-travel health advice could improve the travellers’ risk perception (Wynberg et al., 2013). In general, the travellers in our study underestimated the risk of getting an infectious disease. The low risk awareness resulted in poor precautionary behaviour, which could possibly have been improved by extended pre-travel health advice. Such advice should contain information on transmission routes of antibiotic-resistant bacteria, which for ESBL-producing bacteria often are through food or contaminated water, for example, from vegetables and fruit washed in water contaminated with faecal bacteria (Gaarslev & Stenderup, 1985; Kennedy & Collignon, 2010; Meyer, Gastmeier, Kola, & Schwab, 2012; Struelens et al., 2010), or by inadequate hand-washing (Folkhälsomyndigheten, 2013). Some of the travellers in our study used antibiotics for travellers’ diarrhoea, sometimes without a diagnosis, and sometimes without knowing what drug they were using. As the use of antibiotics for travellers’ diarrhoea increases the risk for acquiring ESBL (Kantele et al., 2015; Mölstad, Cars, & Struwe, 2008), there is a need for improved information for travellers to avoid antibiotics on this indication during the trip.

The Swedish Public Health Agency (PHA) has national responsibility for public health issues. The agency promotes good public health, including infectious disease prevention. Strama (the Swedish Strategic Programme against Antibiotic Resistance), in association with the PHA, provides the public with frequent information about antibiotic use and its effects on the emergence of antibiotic-resistant bacteria (Mölstad, Cars, & Struwe, 2008). Little attention, however, is given to how carriership of antibiotic-resistant bacteria can be prevented while travelling. In this study, the travellers put themselves at risk of acquiring antibiotic-resistant bacteria, and for a majority of them this was because of a lack of knowledge of antibiotic resistance and the risks involved. The risk of getting infected with antibiotic-resistant bacteria is not only personal, but also affects the overall level of resistance in one's home country and thus globally. It is possible to be a carrier without showing any symptoms and there is the possibility of unwittingly spreading carriership to family members, within health care, and in one's immediate environment (Kantele et al., 2015). This also demonstrates that more specific information about the risks and necessary prevention measures for foreign travel to high-risk areas of antibiotic-resistant bacteria is needed. Although none of the participants in our study were “medical tourists”, this type of travel is a growing concern. Travelling abroad for healthcare has increased rapidly, and common destinations are India, Thailand, China, Mexico, and the Middle East (Chen & Wilson, 2013; Nelson, 2011). These areas have a high rate of antibiotic-resistant bacteria, and individuals returning as carriers could place others at risk (Hawkings, Wood, & Butler, 2007). Information from national authorities also about “medical tourism” and its consequences and risks is therefore needed.

How to communicate pre-travel advice about travel risks has been given little attention, as has the travellers’ interpretation about risk information. The fact that some travellers remain healthy without any preventive measures, and others become ill despite following advice, could be confusing. It is a paradox that could result in non-compliance to pre-travel advice. There are also travellers for whom taking risks may be an important part of having an adventure during the trip. The aim of pre-travel advice might sometimes be a behavioural change, and to achieve that goal it might be important to focus on the individual traveller and the destination of the travel (Angelin, Evengård, & Palmgren, 2014; Leder, Steffen, Cramer, & Greenaway, 2015; Noble, Willcox, & Behrens, 2012) Authorities may have difficulty ensuring that travel information reaches the public, but here media can act as a conduit, as in the case of medical science informing the public (Madle, Kostova, Mani-Saada, Weinberg, & Williams, 2004). Other ways to communicate information could be to use modern technology such as smart phones or internet with Facebook, blogs, and text messages. The experience from other travellers could also be useful for compliance to pre-travel advice (Dennisson, Morrison, Conway, & Yardley, 2013; Noble, Willcox, & Behrens, 2012).

Grounded theory is a method for new areas of research, and in terms of this study there has been no scientific publication yet about Swedish travellers’ knowledge of antibiotic resistance and risk-taking during trips abroad. It is also a method that is suitable for studying the social processes at issue in this case. In the theoretical model of the coding process (Figure 2), it is clear that the lack of knowledge about antibiotic-resistant bacteria leads to consequences for the travellers. They were not aware of the risks and with the consequences they exposed themselves to risk situations without knowing it. The core category that emerged described that it is important for travellers to have enough knowledge about antibiotic resistance to avoid those risks. Why is this new knowledge important? Because it is possible to prevent becoming a carrier of antibiotic-resistant bacteria during travels abroad. Everyone could take actions to avoid transmission and take the responsibility not to spread this to others as this is one of our biggest public health problems globally. The purpose of grounded theory methodology is to generate theory through the analytical process of coding and constant comparison. In the methodology of grounded theory, the authors and first constructors Glaser and Strauss argued that the researcher should be “free from theory,” that is free from preconceptions (Glaser & Strauss, 1967), but no researchers are without their own history and background. Preconceptions are not the same as bias, unless the researcher fails to mention them. The main author has many years of experience as an infection control nurse, and that may of course have had some impact on both interviews and analyses. Another limitation to be taken into consideration is that the majority of those in the study group are women. There is no clear explanation for this, but it could be because women are more conscientious in visiting a vaccination clinic before a journey or that they are more willing to participate in research studies. It is difficult to say in what way this may have influenced the results. Another limitation is that the sample is limited. However, the results are transferable to people in similar circumstances, although not necessarily to others. The study group consisted of travellers from a big city, and there may be differences in knowledge and risk assessment when compared with other parts of Sweden. Finally, Sweden still has a relatively low prevalence of antibiotic-resistant bacteria, which may also affect the perception of risk before trips abroad. Thus, there is a need for further research into travellers’ risk behaviour as a basis for the development of better pre-travel advice as regards antibiotic resistance.

## Conclusions

With the increase of global business, leisure travel, and medical tourism, there is a strong need for improved pre-travel advice about the risks of acquiring antibiotic-resistant bacteria during foreign travel.

- Information about the risk of acquiring antibiotic-resistant bacteria during travel to foreign countries should be disseminated through different media, for example, through cross-community campaigns, videos, smart phones, and the internet, where national and regional authorities have an important role to play.
- These campaigns should be expanded to provide more specific information about the risks and prevention measures for travel to countries with a high risk of acquiring antibiotic-resistant bacteria, including what to eat and drink, the importance of hand-washing to prevent the spread of faecally/orally transmitted diseases, and to avoid antibiotics for travellers’ diarrhoea.
